# Eating Disorders in Adolescents With Dental Braces: A Case Report

**DOI:** 10.7759/cureus.57845

**Published:** 2024-04-08

**Authors:** Nawaf H Al Shammary

**Affiliations:** 1 Department of Preventive Dentistry, College of Dentistry, University of Ha'il, Ha'il, SAU

**Keywords:** orthodontics brackets, case report, dental braces, adolescent, eating disorders

## Abstract

Orthodontic treatment during adolescence can contribute to the development of problematic eating disorders. This case study presents the situation of a 12-year-old male patient who experienced a prolonged period of dietary limitation, showed signs of chronic illness, and underwent a significant weight loss because of wearing dental braces. These circumstances triggered the emergence of atypical eating behaviors and complicated the therapeutic process. A case report highlights the effectiveness of conducting psychological evaluations for patients with braces who experience significant weight loss to address possible eating disorders. It also addressed the effectiveness of psychoeducation supportive therapy and nutritional rehabilitation for establishing regular eating patterns during orthodontic treatment. This case also illustrates the significant role of parents in offering emotional support and enhancing professional care. However, conducting extensive longitudinal studies is imperative to fully explore the relationship between orthodontic treatment and eating disorders.

## Introduction

Eating disorders are psychiatric disorders characterized by abnormal eating behaviors, frequently accompanied by psychological disturbances related to weight and body image [1]. Individuals diagnosed with eating disorders have a mortality rate as high as 25%. Moreover, the prevalence of eating disorders is higher in girls than in males [2]. Bulimia nervosa and anorexia nervosa are the most common forms of eating disorders [3]. Anorexia nervosa is generally characterized by a persistent fear of weight gain, resulting in limited food intake, even being below the normal weight range [4]. On the other hand, bulimia nervosa is recognized by episodes of excessive eating, frequently accompanied by self-induced vomiting, to maintain a low body weight [5]. Both bulimia nervosa and anorexia nervosa are highly recognized as serious illnesses that pose a substantial risk to the whole physical and mental well-being of patients, being more frequent during adolescence and the period for starting orthodontic treatment [2].

Previous research has indicated that the prevalence of abnormal eating disorders among female students in Thailand ranged from 9% to 10% [6]. About 15.9% of Thai medical students were found to exhibit abnormal attitudes and behavior toward eating [6]. Another study noted a higher prevalence of anorexic behavior (21.7%) among female healthcare students at the Aga Khan University Hospital, Karachi, Asia [7]. Researchers have recommended a thorough evaluation of medical students showing abnormal eating attitudes and behaviors including an assessment of their mental health, quality of life, academic performance, and career prospects [6].

Research conducted in Asian countries has highlighted the association between weight stigma, abnormal eating attitudes and behaviors, and mental health outcomes. In Saudi Arabia, a robust significant correlation was observed between weigh self-stigma scores on measures of depression, anxiety, and stress scale scores among youths [8].

Individuals with eating disorders often encounter various oral manifestations, including dentinal hypersensitivity, enamel erosion, dental caries [9], malocclusion, enamel demineralization, and xerostomia, with tooth erosion being the most commonly reported manifestation [9]. Orthodontic treatment is a dental procedure that aims to improve a person's function and appearance by utilizing dental braces to gradually align and straighten teeth [1].

Over the last 20 years, there has been a steady increase in the proportion of children undergoing orthodontic treatment using fixed appliances, such as braces [10]. Seeking orthodontic intervention during childhood and adolescence aims to improve dental appearance and promote psychological welfare. Adolescence is a period marked by significant changes in physical, social, and psychological aspects, where the first indications of psychiatric issues frequently emerge [3]. However, psychological and social issues of adolescent patients are often overlooked during orthodontic treatment [11-13]. Patients may encounter varying degrees of pain, discomfort, tension, and soreness of pain during the initial stage of orthodontic treatment [14]. Typically, orthodontic treatment can affect a patient's oral health, quality of life, eating habits, and quality of consumed food within the initial three to six months of treatment [15].

While some aspects of the relationship between eating disorders and orthodontics are well understood, there are still areas that require further understanding. For example, individuals with bulimia nervosa may experience tooth erosion, cavities, and tooth sensitivity due to frequent vomiting. These can impact the structure of teeth, affecting the success of orthodontic treatment [2]. Moreover, severe calorie restriction in anorexia nervosa can lead to malnutrition, which can negatively affect the gingival and bone health, causing delays in tooth movement during orthodontic treatment [9].

It is essential for the orthodontist to be aware of the case's history of eating disorders when designing treatment plans, and possible symptoms of these conditions, know the appropriate time and place to seek advice, and understand how these disorders could affect the outcome of orthodontic treatment [10]. There is still much to be understood about the best approaches to addressing these challenges, with the goal of improving treatment strategies and outcomes for patients with eating disorders undergoing orthodontic treatment.

This case report investigates a young individual who experienced prolonged dietary limitation due to the use of dental braces, resulting in the emergence of atypical eating behaviors and serious therapeutic complications. In addition, it outlines the rehabilitation strategies that were implemented to support the patient and assist him in establishing healthy eating habits.

## Case presentation

A 12-year-old male patient arrived at the emergency room of the dental clinic displaying severe fatigue and widespread abdominal pain persisting for the past eight days. The patient exhibited signs of chronic disease and a significant decrease in body weight, measuring 29 kg with a height of 149 cm, resulting in a body mass index (BMI) of 11.23 kg/m^2^. At the time of hospitalization, the patient had not yet experienced menarche. Laboratory tests revealed the presence of both anemia and leukocytosis, with a total leucocyte count of, 13,000/μL. The red blood cell count was 383×10^4^/μL, hemoglobin measured 8.4 g/dL, and hematocrit level was 29.2%. The total protein concentration was 6.9 grams per deciliter, with albumin level at 3.1 grams per deciliter. The molar concentration of sodium to potassium was 128:2.5 milliequivalents per liter, while the molar concentration of calcium to phosphorus to magnesium was 7.9:3.9:1.98 milligrams per deciliter (Table 1). An abdominal computed tomography scan did not show any notable abnormalities related to the abdominal pain. Consequently, he was admitted to the pediatric hospital for further assessment of his physical status. His mother agreed to additional mental assessment and a non-pharmacological intervention to address the eating disorder. The patient exhibited no signs of depression, anxiety, distorted body image, or concerns about weight gain. During this period, he exhibited all the necessary characteristics for the disorder.

**Table 1:** The case charactersistics and findings

Case character	Findings
Age	12 years and 2 months
Time from getting braces	12 months
Body weight	Kg
Before braces	37
Admission	29
Discharge	32
Body Mass Index BMI	Kg/m2
Before braces	19.2
Admission	11.23
Discharge	12.1
Pathognomonic laboratory findings	Mild leukocytosis
Clinical Documentation Improvement CDI	
Admission	4
Discharge	1
The 26- item Eating Attitude Test (EAT-26)	
Admission	6
Discharge	1
Medications for psychological disorder	None
Length of total hospitalization	24 days

Avoidant/Restrictive Food Intake Disorder (ARFID) as outlined in the fifth edition of the Diagnostic and Statistical Manual of Mental Disorders (DSM-5) by Kocsis [13], used to be classified as an Eating Disorder Not Otherwise Specified (EDNOS). The patient's history indicated that the patient used orthodontic braces for a year to correct his dental misalignment. Before wearing braces, the patient maintained a well-balanced diet with appropriate portion sizes, and his BMI of 19.2 kg/m^2^ was normal for his age and gender. The patient documented an 8 kg weight loss due to the braces, with 4.5 kg lost in the last four months.

Initially, during orthodontic treatment, the patient adopted the practice of fragmenting their meals into smaller portions to facilitate chewing and avoid consuming hard foods to alleviate the discomfort from the braces, which caused severe pain, while eating, leading to a fear of biting and chewing.

Subsequently, the patient began avoiding school meals and gradually reduced food intake at home. Despite the braces reducing pain for a year, the patient persisted in restricting food due to feelings of emptiness. The patient attributed the changes in eating habits to discomfort from dental braces, which led his parents to link low food intake to oral pain. The patient received psychoeducation supportive therapy and nutritional rehabilitation in the pediatric ward to address eating issues. Cognitive behavioral therapy (CBT) is a proven effective approach for various mental health conditions, including eating disorders [16]. The Psychiatric Department at the hospital recommended 18 therapeutic sessions of CBT to help identify and change maladaptive thinking and behavior patterns. Furthermore, nutritional rehabilitation focused on correcting health problems caused by malnutrition, preventing dieting, or binging, and establishing regular eating patterns through taking three meals a day with snacks daily.

After 24 days of treatment and an increase in calorie intake, the patient was prescribed 5 mg of mirtazapine to boost his appetite and received extra psychological support. Education on eating disorders was given, with a treatment plan that required a minimum daily intake of 1,800 calories. Furthermore, the patient began incorporating soft noodles and semi-liquid meals in small portions to help alleviate their oral pain.

The patient's elevated white leukocyte counts and decreased erythrocyte count returned to normal levels. Specifically, the count of white blood cells was 14,000 per microliter, red blood cells was 430 times 10 to the power of 4 per microliter, the hemoglobin level was 12.8 grams per deciliter, and the hematocrit level was 34.9%.

The patient gained 3 kg upon discharge after achieving physical stability. However, the patient showed a lack of willingness to continue receiving medical care and declined further psychiatric assessments, despite undergoing them upon admission, leading to discharge with schedules for nutritional education and regular follow-up appointments within the Psychiatric Department in the hospital.

## Discussion

Eating disorders are among the most common, prevalent, and curious psychological diseases, affecting over 14 million people, including three million children and adolescents. This places them second to anxiety disorders, which affect 301 million people, including 58 million children and adolescents [17]. Typically appearing during adolescence [9], a critical phase for the development of cognitive functions, body image perception, and susceptibility to peer influence [18]. These disorders can have medical consequences on various physiological systems and can persist into adulthood [19].

Researchers have provided evidence about the comorbidity between eating disorders and other psychiatric disorders, indicating an increased risk of psychiatric disorders in those with eating disorders, and an increased risk of eating disorders in individuals with various psychiatric disorders [20].

Eating disorders arise from an intricate interaction of multiple factors, encompassing both biological and sociocultural elements. Presently, people are exhibiting unusual eating behaviors and deliberately skipping meals to alleviate the discomfort caused by braces [21]. Nevertheless, they did not express concerns about their weight or appearance [22]. Despite the tendency for psychological issues to remain hidden during adolescence, these individuals maintained consistent eating patterns and gradually gained weight until undergoing orthodontic treatment [23]. However, as the orthodontic treatment progressed [24], they developed unusual eating habits that persisted long after the dental discomfort subsided. Existing evidence suggests that orthodontic treatment-related eating problems could increase the likelihood of adolescents developing maladaptive coping strategies, potentially initiating or sustaining eating disorders [25].

Recent research has shown that some individuals even start orthodontic treatment before developing anorexia nervosa [26]. Therefore, evaluating individuals experiencing oral discomfort for unintended weight loss is crucial [18].

Although the Diagnostic and Statistical Manual of Mental Disorders, Fourth Edition, Text Revision (DSM-IV-TR) does not provide specific diagnoses for these cases [24], they can accurately be categorized under the newly introduced ARFID diagnostic category in the DSM-5 [27]. The individuals' regular and restricted eating behaviors warranted a diagnosis of Anorexia Nervosa, specifically the subtype characterized by restriction [27]. Despite lacking overt fear or avoidance related to weight gain [28], the patient tended to minimize the seriousness of his weight loss during the assessment, potentially indicating a diagnosis of anorexia nervosa. Hence, it is crucial to observe subtle indicators that could hint at an underlying eating disorder.

Currently, it is well-known that patients with braces often exhibit atypical eating patterns to avoid food to cope with the discomfort caused by orthodontic treatment [3]. These patients have not expressed any concerns about their body weight or physical appearance. Concealing psychiatric diseases during adolescence is a prevalent phenomenon [5], yet the psychosocial challenges faced by adolescent patients are often overlooked in the context of orthodontic treatment [8]. Several studies have investigated the association between psychiatric disorders and dental interventions. However, it is important to note that these studies have primarily focused on older age groups or pre-surgical psychological assessments [7,11,14].

Eating disorders have significant clinical implications for health providers and orthodontists involved in providing healthcare to those affected. In dental practice, dentists may observe signs such as tooth erosion dry mouth, and hypersensitivity [9].

In this case, understanding the clinical implications of eating disorders is essential for a comprehensive approach to treatment and support. Psychologists play a crucial role in providing diagnosis and treating eating disorders, as these disorders are associated with other mental health issues such as depression and anxiety [8]. Furthermore, the impact of an eating disorder on family life has been documented [29]. Researchers have addressed the importance of understanding the parent's perspective of having a child with an eating disorder [29]. This case also indicated that parents and caregivers have a critical role in the recovery process, providing emotional support, observing behavior, and enhancing professional treatment when required.

## Conclusions

In summary, orthodontic braces can sometimes lead to eating challenges causing anemia, and electrolyte imbalances. These difficulties arise from the need to carefully fragment meals by breaking them into smaller portions before eating and chewing cautiously to avoid hard foods.

In this case, by following a well-balanced diet under hospital supervision and receiving psychometric assessment, the patient effectively managed these eating concerns. CBT, a type of psychoeducation, displayed potential in supporting a patient with eating disorders and guiding him toward establishing healthy eating habits. Additionally, nutritional rehabilitation played a significant role in assisting the patient in maintaining a healthy eating pattern during orthodontic treatment. Moreover, this case hints that parents and caregivers can offer emotional support to complement professional dental care. Nonetheless, it is vital to conduct extensive longitudinal studies to fully explore the relationship between orthodontic treatment and eating disorders. This rigorous research is crucial for improving understanding and clinical outcomes.
